# Diabetic Retinopathy among Patients with Prediabetes Attending the Outpatient Department of Ophthalmology in a Tertiary Eye Care Centre: A Descriptive Cross-sectional Study

**DOI:** 10.31729/jnma.8118

**Published:** 2023-04-30

**Authors:** Arjun Shrestha, Rinkal Suwal, Sikshya Adhikari, Nirsara Shrestha, Biju Shrestha, Bijay Khatri

**Affiliations:** 1 Department of Ophthalmology, BP Eye Foundation, Hospital for Children, Eye, ENT, and Rehabilitation Services, Madhyapur Thimi, Bhaktapur, Nepal; 2 Department of Optometry, BP Eye Foundation, Hospital for Children, Eye, ENT, and Rehabilitation Services, Madhyapur Thimi, Bhaktapur, Nepal; 3 Department of Physiology, Kathmandu Medical College, Sinamangal, Kathmandu, Nepal; 4 Academic and Research Department, BP Eye Foundation, Hospital for Children, Eye, ENT, and Rehabilitation Services, Madhyapur Thimi, Bhaktapur, Nepal

**Keywords:** *diabetic retinopathy*, *prediabetes*, *prevalence*

## Abstract

**Introduction:**

Diabetic retinopathy is a specific microvascular ocular complication associated with diabetes. However, retinopathy has also been reported in people with prediabetes. The study aimed to find out the prevalence of diabetic retinopathy among patients with prediabetes attending the outpatient Department of Ophthalmology in a tertiary eye care centre.

**Methods:**

A descriptive cross-sectional study was conducted among patients with prediabetes attending the outpatient Department of Ophthalmology in a tertiary eye care centre from 1 January 2022 and 30 April 2022. Ethical approval was obtained from the Ethical Review Board (Registration number: 594/2021 P). All patients had their eyes dilated and examined under the slit-lamp with a 90 D convex lens or indirect ophthalmoscopes with a 20 D lens to find retinopathy. All patients aged 40 to 79 years with intermediate hyperglycemia were included in the study. Convenience sampling was used. Point estimate and 95% Confidence Interval were calculated.

**Results:**

Among 141 patients with prediabetes, diabetic retinopathy was found in 8 (5.67%) (1.859.49, 95% Confidence Interval). Among which all patients 8 (5.67%) had mild non-proliferative diabetic retinopathy. Among patients with retinopathy, all 8 (5.67%) were obese, 3 (37.50%) were hypertensive, 5 (62.50%) patients had intermediate hyperglycemia for more than 6 months, and 2 (25%) had a family history of diabetes mellitus.

**Conclusions:**

The prevalence of diabetic retinopathy in prediabetes patient was found to be higher than the other studies conducted in similar settings.

## INTRODUCTION

Diabetic retinopathy (DR) is a specific microvascular ocular complication associated with diabetes.^1^ However, retinopathy has also been reported in people with prediabetes.^2^ Prediabetes is a health condition with raised blood glucose levels above the normal range and below the diabetes diagnostic threshold.^3^

The estimated global prevalence of intermediate hyperglycemia is as high as 10.6% and 8.5% in Nepal.^3,4^ The burden of prediabetes is an alarming health issue as they signify a higher risk of the future development of diabetes, increased risk of cardiovascular disease, with early forms of nephropathy, chronic kidney disease, small fibre neuropathy, diabetic retinopathy, and increased risk of macrovascular disease.^5,6^ DR is the foremost reason for vision impairment and blindness within the economically remunerative working-age population globally.^7^ Early detection of retinopathy in prediabetes will be a more secure strategy to prevent vision loss.

The study aimed to find out the prevalence of diabetic retinopathy (DR) among patients with prediabetes attending the outpatient Department of Ophthalmology in a tertiary eye care centre.

## METHODS

A descriptive cross-sectional study was conducted among patients with prediabetes attending the outpatient Department of Ophthalmology at Hospital for Children, Eye, ENT, and Rehabilitation Services (CHEERS), from 1 January 2022 to 30 June 2022. Ethical approval was taken from the Institutional Review Committee (Reference number: 594/2021 P). All patients aged 40 to 79 years with prediabetes were included in the study. Patients with known diabetes and glaucoma, newly diagnosed diabetes, on any medication for diabetes, or women with gestational diabetes were excluded from the study. A convenience sampling method was used. The sample size was calculated by using the following formula:


$$\begin{array}{ll} \text{n} & ={\text{Z}}^{2}\times \frac{\text{p}\times \text{q}}{{\text{e}}^{\text{2}}}\\ & =1.96^{2}\times \frac{0.50\times 0.50}{0.09^{2}}\\ & =119 \end{array}$$

Where,

n = minimum required sample size
Z = 1.96 at 95 % Confidence Interval (CI)
p = prevalence taken as 50% for maximum sample size calculation
q = 1-p
e = margin of error, 9%

The calculated sample size was 119. After adding 10% non response rate, the total sample size was 132. However, we included 141 patients for this study.

In this study, patients with laboratory reports of impaired glucose tolerance values between 140199 mg/dl and impaired fasting glucose between 110-124 mg/dl in the past four weeks were defined as patients with prediabetes.^3^ Study participants underwent anthropometric measurements and measurement of blood pressure at the health promotion unit of CHEERS under the standard protocol developed earlier.^8^ The body mass index (BMI) was classified according to South Asian population cutoff points with BMI between 23 and 24.9 kg/m^2^ classified as overweight and BMI greater than 25 kg/m^2^ as obese.^9^ The patients with blood pressure ≥130 and/or ≥80 mmHg were classified hypertensive,^10^ including those under medication for lowering blood pressure.

The study permission was taken from the study site, CHEERS, and written informed consent was obtained from all the participants. In the case of an illiterate patient, the assent was taken from the accompanying friend. The patient's eye was dilated with tropicamide 5%, phenylephrine 2.5%, and tropicamide 5% only if the patient was hypertensive. A consultant retina specialist performed the slit-lamp examination with a 90 Dioptre (D) convex lens or indirect ophthalmoscopes with a 20 D lens to find retinopathy. The minimum criterion for diagnosis of DR was the presence of at least one definite lesion (microaneurysm) in any field of the retina in either eye. The final diagnosis for each study participant was determined from the level of DR of the worse eye using the International Clinical Diabetic Retinopathy scale.^11^ Diagnosis of mild, moderate and severe mild NPDR, and Proliferative diabetic retinopathy was diagnosed based on Early Treatment Diabetic Retinopathy Study (ETDRS) diabetic retinopathy severity scale as per in previous study.^12^ A trained ophthalmic assistant conducted face-to-face interviews for socio-demographic variables-related questions.

Data were entered in Microsoft Excel Version 2010 and analyzed using IBM SPSS Statistics version 26.0. Point estimate and 95% CI were calculated.

## RESULTS

Among 141 patients with prediabetes, 8 (5.67%) (1.85-9.49, 95% CI) had DR. Among which all 8 (100%) patients had mild NPDR. Among patient with mild NPDR, 6 (75%) had mild NPDR in the right eye, and the 2 (25%) had mild NPDR in the left eye.

Among patients with mild NPDR, 5 (62.50%) of patients were from the 60-79 years old age group. The mean age of all patients with mild NPDR retinopathy was 60.88±11.93 years and that of male and female, were, 60.50±14.27 years, and 60.25±11.33 years respectively. Only 2 (25%) of mild NPDR retinopathy patients had attended university education (Table 1).

**Table 1:** Socio-demographic characteristics (n= 8).

Characteristics	n (%)
**Age (years)**	
40-59	3 (37.50)
60-79	5 (62.50)
**Gender**	
Male	4 (50)
Female	4 (50)
**Educational status**	
Iliterate	3 (37.50)
School education	3 (37.50)
University education	2 (25)

The mean BMI among mild NPDR retinopathy patients was 29.10±2.04 kg/m^2^, and all were obese according to the BMI classification for the South Asian population. The mean systolic blood pressure (SBP) and diastolic blood pressure (DBP) of diabetic patients with mild NPDR retinopathy were 123.75±7.44 and 80.00±5.35 mmHg, respectively. Among them, 3 (37.50%) were hypertensive (Table 2).

**Table 2:** Distribution of overweight and hypertension (n= 8).

Characteristics	n (%)
**Obesity according to BMI**	
Overweight	-
Obese	8 (100)
Hypertensive	3 (37.50)

Among patients with mild NPDR retinopathy, 5 (62.50%) patients had intermediate hyperglycemia for more than 6 months, and only one-fourth, 2 (25%) had a family history of diabetes mellitus (Table 3).

**Table 3:** Duration of prediabetes and family history of diabetes mellitus (n = 8).

Characteristics	n (%)
**Duration of prediabetes (months)**	
<6	3(37.50)
>6	5(62.50)
Family history of diabetes mellitus	2(25)

## DISCUSSION

The prevalence of DR in prediabetic patients in our study was 5.67%, which is lower than in the neighboring country India, where the prevalence was 6.3%.^13^ Our study's slightly less prevalence could be due to the study in India being conducted in two tertiary care diabetes centers, with a larger sample size and fundus photography for documenting DR lesions. Studies from China in two different municipalities, Shanghai and Chongqing, showed a prevalence of 2.5% and 20.91%, respectively.^14,15^ Studies in Australia,^16^ the USA,^17^ and Germany,^18^ have also reported prevalence of 6.7%, 7.9%, and 8.1%, respectively. These findings suggest that earlier screening for retinopathy in prediabetic conditions should be considered.

All patients (100%) had mild NPDR in our study, which is higher than the neighbouring country India, where the prevalence was 75%.^13^ Studies from China in two different municipalities, Shanghai and Chongqing, showed a prevalence of 2.1% and 100%, respectively.^14,15^ Studies in Australia,^16^ the USA,^17^ and Germany,^18^ have also reported a prevalence of 62.66%, 14.4%, and 92%, respectively. These findings suggest that earlier screening for retinopathy in prediabetic conditions should be considered.

All patients had mild NPDR, and none had severe sight-threatening DR in our study. The study in Chongqing, China, also reported that none had severe sight-threatening DR,^15^ whereas the study in Chennai, India also noted the presence of moderate NPDR among 1.6% of prediabetic patients.^13^ Early Treatment Diabetic Retinopathy Study (ETDRS) has recommended observation for mild and moderate NPDR,^15^ indicating that prediabetic patients need to visit ophthalmologists at least once a year.

All 8 (100%) patients with mild NPDR retinopathy were obese. With similar BMI categorization, it has been reported that the increase in BMI was associated with an increased risk of prediabetes^19^ and positively associated with DR.^20^ Our study also showed that 37.5% of mild NPDR retinopathy patients were hypertensive. A study has shown that treated but poorly controlled and untreated hypertension is associated with any DR.^21^

Our study showed that among patients with mild NPDR retinopathy, 62.5% had prediabetic condition for more than 6 months. Lifestyle intervention may decrease the risk of prediabetes progressing to diabetes for as long as ten years,^22^ and ultimately any DR. To prevent or reduce the progression to severe visual loss in patients with prediabetes, we need to develop appropriate interventions to lower BMI and blood pressure and prevent developing diabetes.

This is a hospital-based study and hence cannot be generalized which was one of the limitations of this study.

## CONCLUSIONS

The prevalence of mild NPDR retinopathy in patients with prediabetes was higher in our study than the other study done in similar settings. Dilated eye examination for retinopathy screening is recommended for patients with prediabetes, along with promoting a healthy lifestyle to control the development of risk factors, diabetes, and other related health complications.
